# Direct and Indirect Antioxidant Effects of Selected Plant Phenolics in Cell-Based Assays

**DOI:** 10.3390/molecules26092534

**Published:** 2021-04-26

**Authors:** Jakub Treml, Petra Večeřová, Petra Herczogová, Karel Šmejkal

**Affiliations:** 1 Department of Molecular Pharmacy, Faculty of Pharmacy, Masaryk University, Palackeho tr. 1946/1, 612 00 Brno, Czech Republic; petra.vece@seznam.cz (P.V.); petra.herczogova@gmail.com (P.H.); 2 Department of Natural Drugs, Faculty of Pharmacy, Masaryk University, Palackeho tr. 1946/1, 612 00 Brno, Czech Republic

**Keywords:** antioxidants, CAA, catalase, glucose toxicity, plant phenolics, superoxide dismutase, Nrf2-ARE

## Abstract

**Background**: Oxidative stress is a key factor in the pathophysiology of many diseases. This study aimed to verify the antioxidant activity of selected plant phenolics in cell-based assays and determine their direct or indirect effects. **Methods**: The cellular antioxidant assay (CAA) assay was employed for direct scavenging assays. In the indirect approach, the influence of each test substance on the gene and protein expression and activity of selected antioxidant enzymes was observed. One assay also dealt with activation of the Nrf2-ARE pathway. The overall effect of each compound was measured using a glucose oxidative stress protection assay. **Results**: Among the test compounds, acteoside showed the highest direct scavenging activity and no effect on the expression of antioxidant enzymes. It increased only the activity of catalase. Diplacone was less active in direct antioxidant assays but positively affected enzyme expression and catalase activity. Morusin showed no antioxidant activity in the CAA assay. Similarly, pomiferin had only mild antioxidant activity and proved rather cytotoxic. **Conclusions**: Of the four selected phenolics, only acteoside and diplacone demonstrated antioxidant effects in cell-based assays.

## 1. Introduction

Oxidative stress is a disturbance of the balance between pro-oxidant and antioxidant states that favors the former. The essence of the pro-oxidant process is the production of reactive oxygen (ROS) and nitrogen species (RNS). ROS include molecules of various structures: for example, oxygen radicals (such as hydroxyl radical ^•^OH or peroxyl radical RO_2_^•^) and strongly oxidizing non-radical substances (such as hydrogen peroxide, H_2_O_2_) [1,2]. ROS production, which leads to DNA damage, protein alteration, or lipid peroxidation, is a known factor in developing various pathological conditions, such as cardiovascular diseases, cancer, neurological disorders, diabetes mellitus, and aging [3].

In addition to these deleterious effects, there is also a positive side to producing ROS. For example, they are produced by phagocytic NADPH oxidase (in oxidative burst), and NO^•^ regulates vascular tone [1,3]. Current understanding uses the concept of “eustress”, a certain level of oxidative stress necessary for cellular life. For example, H_2_O_2_ at nanomolar concentrations serves as a redox signaling molecule, but at supraphysiological concentrations (>100 nM), it damages biomolecules [4].

Two basic groups of antioxidants are usually recognized as providing cellular protection against harmful oxidative stress: (i) direct antioxidants, which undergo redox reactions and scavenge ROS or RNS, and (ii) indirect antioxidants, which may or may not be redox-active and activate the nuclear factor erythroid 2 (NFE2)-related factor 2 (Nrf2) and antioxidant response element (ARE) pathway resulting in antioxidant enzyme expression [4].

Antioxidant activity assays follow the same division. The direct antioxidant effect can be evaluated using a chemical-based assay, such as the 2,2-diphenyl-1-picrylhydrazyl radical (DPPH^•^) scavenging assay or the ferric reducing antioxidant power (FRAP) assay. In general, the mechanisms of these in vitro methods depend on scavenging stable free radicals or reducing ferric ions, respectively. Another approach is to establish the antioxidant capacity under more biologically relevant conditions. These assays are cellular-based and focus on direct scavenging. An example of such a method is the cellular antioxidant activity (CAA) assay, which identifies antioxidants able to prevent ROS (obtained from the decomposition of 2,2′-azo*bis*(2-methylpropionamidine) dihydrochloride (AAPH)) from oxidizing dihydrodichlorofluorescein DCFH_2_ to fluorescent dichlorofluorescein DCF [5].

The indirect antioxidant activity reflects the removal of ROS by enzymes, such as superoxide dismutase—SOD or catalase—CAT, and small thiol molecules, e.g., glutathione (GSH). SOD converts superoxide anion (O_2_^•−^), a byproduct of normal oxygen metabolism, into H_2_O_2_, which CAT then decomposes into water and oxygen [6]. The expression of both enzymes is regulated by ARE, which is activated by Nrf2 [7]. A member of the cap ‘n’ collar (CNC) subfamily of basic region leucine zipper (bZip) transcription factors, Nrf2 is found in an inactive form in the cytosol, bound to Kelch-like ECH-associated protein 1 (Keap1). Upon oxidative stress, Nrf2 dissociates from Keap1 and translocates into the nucleus, activating the ARE [6,8].

The Nrf2 system was, therefore, found to be the target of various indirect antioxidants, and this mode of action has been confirmed for various phenolics, e.g., epigallocatechin-3-gallate [9] or 3-*O*-caffeoyl-1-methylquinic acid [10]. The proposed mechanism of Nrf2 activation is most likely the alteration of the structure of Keap1 because it contains several cysteine thiol residues that function as sensors of cellular redox changes. Thus, oxidation or covalent modification of some of these residues would release Nrf2 and facilitate its accumulation in the nucleus [11].

The main difference between the two approaches is that the direct measurement is quite rapid, whereas the indirect effect requires more time because it entails the biosynthesis of new proteins [5]. A third approach combines direct antioxidant activity and indirect antioxidant effects in a cellular-based assay, with a long incubation time (e.g., 24 h or longer). In these settings, both direct scavenging and indirect activation of Nrf2/ARE may concur to the final effect. An example of this type of method is the glucose oxidative stress protection (GOSP) assay, which creates a condition of hyperglycemia to increase the production of ROS [12,13].

**Figure 1:**
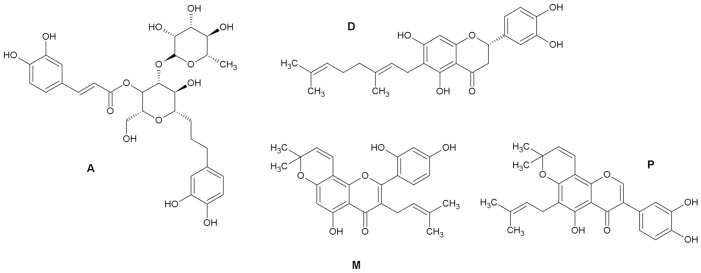
The compounds selected for experiments: acteoside (**A**), diplacone (**D**), morusin (**M**), and pomiferin (**P**).

Dietary phenolics of polyphenols occupy a special place among the antioxidants that occur in plants [14]. The structures of these compounds contain an aromatic ring with one or more hydroxyl groups. This group of natural products is very widely distributed in the plant kingdom, with more than 8000 phenolic structures currently known [15]. Many of these compounds possess direct or indirect antioxidant activity [6]. We selected four plant phenols that had shown some degree of in vitro antioxidant activity [16,17,18,19] for further testing in cell-based assays (Figure 1). Acteoside (**A**) is a caffeoyl phenylethanoid glycoside obtained from *Paulownia tomentosa*, as is the geranylated flavanone diplacone (**D**) [17,20]. Morusin (**M**) was selected as an example of the prenylated flavones obtained from *Morus alba*, and pomiferin (**P**), a prenylated isoflavone, comes from *Maclura pomifera* [18,21]. Here, we aimed our study at testing the direct scavenging or indirect modulation of expression of some antioxidant enzymes by these typical representatives of natural phenolics.

## 2. Results and Discussion

### 2.1. Antiproliferative Activity

The direct scavenging effect and ability to modulate antiradical defense in a model cellular system of the test compounds were analyzed. Before carrying out antioxidant assays in cellular systems, it was necessary to determine the concentrations above which the test compounds became cytotoxic. A model assay employing THP-1 monocytes was used. Diplacone had previously been shown to be non-toxic at a concentration of 10 µM when incubated with THP-1 cells [22]. It had also been found that the half-maximal inhibitory concentration (IC_50_) of morusin for the THP-1 cells is 24.3 µM [23,24].

**Figure 2:**
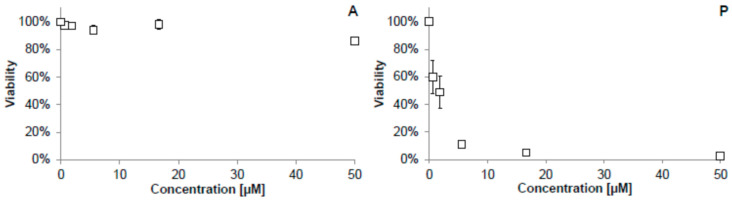
The antiproliferative activity of the test compounds after 24 h of incubation with the THP-1 cell line, measured using a WST-1 kit: (**A**) acteoside; (**P**) pomiferin. The viability was calculated as a percentage of the control cells treated only with DMSO.

The cytotoxic (antiproliferative) activity of the other two test compounds, acteoside and pomiferin, for the THP-1 cell line could not be found in the literature, and it was, therefore, measured using a WST-1 assay kit. As shown in Figure 2, acteoside influenced the viability of THP-1 cells only slightly, even at the high concentration of 50 µM. The information published about cytotoxic effects observed for acteoside is quite inconsistent. Lee et al. measured the cytotoxicity of this compound using the MTT assay on HL-60 human promyelocytic leukemic cells and found the IC_50_ value after a 24 h incubation to be approximately 30 µM [25]. On the other hand, Sgarbossa et al. claimed to find no cytotoxic effect on the immortalized human keratinocyte cell line HaCaT using the MTT assay at a concentration of 200 µM even after 72 h of incubation [26]. Similarly, Nam et al. and Speranza et al. reported no cytotoxic effect on the THP-1 cell line at concentrations of 16 µM and 100 µM, resp. The only difference from our experiment was using the MTT assay, whereas we employed the WST-1 test [27,28].

Pomiferin greatly reduced the viability of THP-1 cells, with an IC_50_ of 1.0 µM (Figure 2), suggesting that it may possess antitumor activity because THP-1 cells are cancer-derived. Our conclusion is consistent with other results. Son et al. found that pomiferin inhibited histone deacetylase (HDAC), an enzyme involved in cell proliferation, and thus may reduce the proliferation of tumor cells. This was confirmed by further experiments with the MTT assay, in which pomiferin inhibited the growth of several human tumor cell lines with IC_50_ ranging from 1 to 5 µM [29]. Similarly, Yang et al. demonstrated the selective antiproliferative activity of pomiferin against the tumorigenic breast epithelial cell line MCF-7 (IC_50_ = 5.2 µM) [30]. Both articles also compared the antiproliferative activity of pomiferin on normal, non-tumor cells—SON et al. employed primary human hepatocytes that were affected much less (IC_50_ = 123 µM) [29]. Yang et al. proved a limited toxicity toward non-tumorigenic breast epithelial cells (MCF-10A) [30].

### 2.2. Cellular Antioxidant Activity (CAA) Assay

After the cytotoxicity had been evaluated, the overall antioxidant activity of the test compounds was analyzed using the CAA assay. CAA measures the ability of compounds to prevent AAPH-generated peroxyl radicals from forming fluorescent DCF in THP-1 cells. AAPH gradually decomposes into carbon-centered radicals that then react rapidly with O_2_ to give ROO^•^ radicals [31]. The compounds were tested at a nontoxic concentration of 5 µM. The toxicity of pomiferin was not considered problematic because a short incubation time was used for this assay (2 h).

**Figure 3:**
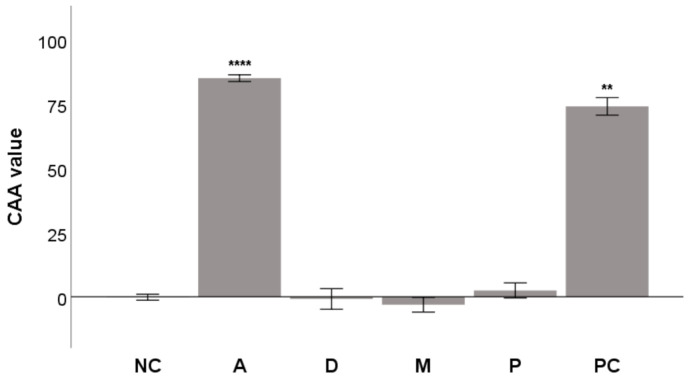
The antioxidant activity of acteoside (**A**), diplacone (**D**), morusin (**M**), and pomiferin (**P**) at a concentration of 5 µM in a CAA assay in THP-1 cells. Quercetin at a concentration of 5 μM was used as a positive control (PC). DMSO, the solvent used for both the test compounds and quercetin, was added as the negative control (NC). The results are expressed as the mean ± SEM for two independent experiments measured in triplicate and are statistically compared to NC (** *p* < 0.01, and **** *p* < 0.0001).

As seen in Figure 3, the most active compound was acteoside, with a CAA value of 85.1 ± 0.7 and activity greater than that of quercetin, the positive control (n.s. difference). The activity of both acteoside and quercetin was significantly higher than that of DMSO, the negative control (NC; Figure 3). The ability of acteoside to scavenge radicals directly has been reported in the literature. Koo et al. showed its DPPH^•^ and NO^•^ scavenging activities [16]. Similarly, Siciliano et al. measured the ability of acteoside to scavenge the radical cation 2,2′-azino-*bis*(3-ethylbenzothiazoline-6-sulfonate) (ABTS^•+^) using the Trolox equivalent antioxidant capacity (TEAC) assay [32]. Further, Li et al. demonstrated the scavenging activity of acteoside in the FRAP and cupric reducing antioxidant capacity (CUPRAC) assays [33]. Moreover, Lee et al. proved the ability of acteoside to scavenge ^•^OH and O_2_^•−^ [34]. The advantage of CAA is that the radical scavenging effect of a compound is measured only inside the cells because after de-esterification DCFH stays inside the cells [35]. It also confirms the ability of acteoside to cross the cell membrane, as shown by Koo et al., who found that acteoside decreased the lipid peroxidation and neurotoxicity of glutamate in cortical cell cultures [16].

Pomiferin showed mild antioxidant activity. It has been reported to be a scavenger of DPPH^•^ and O_2_^•−^ [18,36,37]. Bozkurt et al. also observed that a 300 mg/kg dose of pomiferin administered to rats significantly reduced the lipid peroxidation induced by indomethacin in their stomachs. Lipid peroxidation was measured by determining the levels of malondialdehyde (MDA) [38]. Similarly, Hwang et al. showed the antioxidant activity of the large quantity of pomiferin present in Osage orange extract [39].

Interestingly, neither diplacone nor morusin demonstrated any antioxidant effect in the CAA assay, as seen in Figure 3. Diplacone had previously shown DPPH^•^ scavenging activity and was the most active of the geranylated flavonoids extracted from *P. tomentosa* [17]. Diplacone has shown activity in other antioxidant methods—scavenging of ABTS^•+^, O_2_^•−^, HClO, and inhibiting the plasmid DNA oxidative damage caused by the Fenton reaction [36,40,41]. Similarly, Moon et al. showed that incubation cells of the human lymphoblastoid cell line AHH-1 with diplacone and exposing them to γ-radiation protected them from oxidative stress and DNA damage [42]. However, J774A.1 cells incubated for 30 min with diplacone produced almost double the amount of ROS, measured as DCF, as was produced by untreated cells. Although the difference did not appear to be statistically significant, it demonstrated a mild pro-oxidant effect for diplacone [36]. This result is following the conclusion published by Malaník et al. that a crucial structural element for activity in the CAA assay is the 5,7-*m*-dihydroxy arrangement of the flavonoid ring A, with no substituent at C-6. Diplacone a has geranyl moiety at C-6, and its activity in CAA is, therefore, reduced [43].

Hošek et al. have reported that cudraflavone B, a compound structurally similar to morusin, scavenges HClO [36]. On the other hand, incubating this compound with J774A.1 cells alone for 30 min tripled the production of ROS, measured as DCF, compared to untreated cells [36]. However, contradictory results reported in the literature have found morusin reducing the production of ROS in cell cultures. Cheng et al. reported that morusin reduced 12-*O*-tetradecanoylphorbol-13-acetate (TPA)-mediated production of ROS in a mouse epidermal JB6 P^+^ cell model [19]. Lee et al. reported a decrease in NO^•^-induced cell death in neuroblastoma SH-SY5Y cells during incubation with morusin [44]. Similarly, Yang et al. observed that morusin suppresses the production of NO^•^ in RAW264.7 cells caused by lipopolysaccharide (LPS) and interferon-γ [45]. Moreover, finally, Ko et al. found that morusin inhibits the formation of O_2_^•−^ in rat neutrophils stimulated with phorbol myristate acetate (PMA) [46]. The difference between these findings and our result probably stems from using different cell lines and ROS generators. Morusin was obviously better at counteracting TPA, LPS, NO^•^, PMA, and radicals formed from them than radicals generated by AAPH.

### 2.3. Glucose Oxidative Stress Protection (GOSP) Assay

After the CAA assay experiments, acteoside and diplacone were chosen for further evaluation in a GOSP assay. Morusin was not chosen because it showed the lowest values in the CAA assay. Similarly, pomiferin was omitted due to its unfavorable cytotoxicity profile. In the GOSP assay, the cells were exposed to a hyperglycemic condition that increased oxidative stress. The amount of intracellular stress was visualized by the conversion to fluorescent dichlorofluorescein (DCF).

Chronic hyperglycemia is a characteristic condition for diabetes mellitus (DM) that negatively impacts cells and tissues. The toxicity of high levels of glucose manifests itself, especially in the β-cells of the pancreas, where it reduces the secretion of insulin. In other organs, it is responsible for the chronic microvascular complications of DM. The molecular mechanisms of glucose toxicity involve the glycation of proteins via Schiff bases and Amadori compounds that increase ROS production [47]. Overproduction of O_2_^•−^ in the mitochondrial electron transport chain (ETC) increased production of ROS as glucose is the main energy source and fuel for ETC [48].

**Figure 4:**
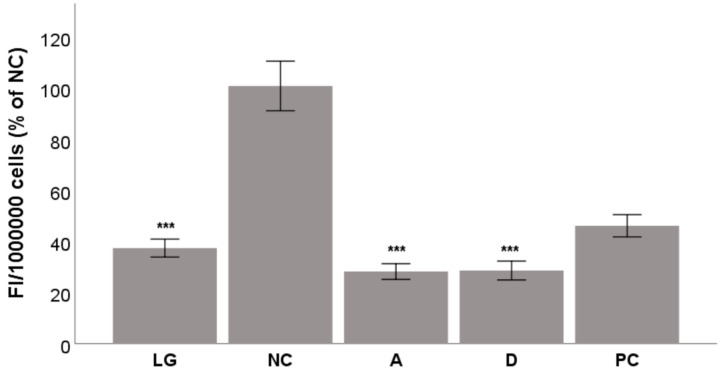
The antioxidant activity of acteoside (**A**) and diplacone (**D**) at a nontoxic concentration of 5 µM in a GOSP assay in HepG2 cells. Quercetin at a concentration of 5 µM was used as a positive control (PC). DMSO, the solvent used for both the test compounds and quercetin, was added as the negative control (NC). Normoglycemic control was achieved using a low glucose medium (LG). All other samples were incubated in a high-glucose medium. The results are expressed as the mean ± SEM for two independent experiments measured in triplicate and were statistically compared to NC (*** *p* < 0.001).

After a 48 h incubation of HepG2 cells in hyperglycemic conditions, both test compounds were able to reduce oxidative stress and the production of DCF to the level of normoglycemia in a statistically significant manner (*p* < 0.001; Figure 4). Both acteoside and diplacone reduced oxidative stress down to 28% of the level of the hyperglycemic condition and were more effective than the quercetin used as a positive control.

El-Marasy et al. have reported that acteoside alleviates oxidative stress in rats with streptozotocin-nicotinamide (STZ-NA)-induced type 2 diabetes [49]. This was observed as reduced levels of malondialdehyde (MDA), a marker of lipid peroxidation. The content of reduced glutathione in the liver was also increased. In addition, acteoside significantly lowered blood glucose levels, glycosylated hemoglobin, and total cholesterol compared to control diabetic rats [49]. Glucose toxicity during hyperglycemia is marked by increased formation of advanced glycation endproducts (AGEs) and greater aldose reductase activity. According to the results of Yu et al., both of these parameters were inhibited by acteoside [50].

Zima et al. described the effects of diplacone administered to rats with alloxan-induced diabetes. While the impact of diplacone on glucose levels was minor, the compound showed a cytoprotective effect on β-cells of the islets of Langerhans, which was confirmed by histopathological analysis. The protection of β-cells correlated with the greater antioxidant activity of diplacone than the other compounds examined [41].

### 2.4. Indirect Antioxidant Activity—Modulation of Antioxidant Enzymes

#### 2.4.1. Protein Expression

Further, it was important to discern whether the test compounds acteoside and diplacone can also protect cells against oxidative stress also by indirect modulation of antioxidant enzymes and not only by the direct scavenging activity shown previously [32,33,36,40,41].

Because the most common representative ROS are O_2_^•−^, H_2_O_2_, and ^•^OH [51], we chose to evaluate antioxidant enzymes that deal with them, namely CAT, SOD1, and SOD2. We also evaluated the expression of another protein—Nrf2, a transcription factor involved in the antioxidant response of cells. We incubated acteoside and diplacone with THP-1 cells and observed their influence on the level of protein expression.

**Figure 5:**
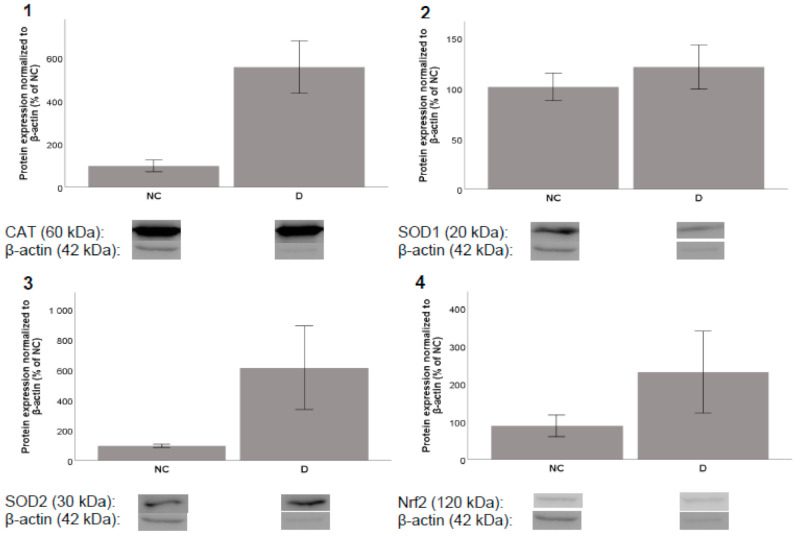
Effects of diplacone (**D**) at a concentration of 2.5 µM on the expression of (**1**) CAT, (**2**) SOD1, (**3**) SOD2, and (**4**) Nrf2 after 8 h incubation with THP-1 cells. DMSO was used as the solvent and was added as the negative control (NC). The results are expressed as the mean ± SEM and were measured in triplicate.

**Figure 6:**
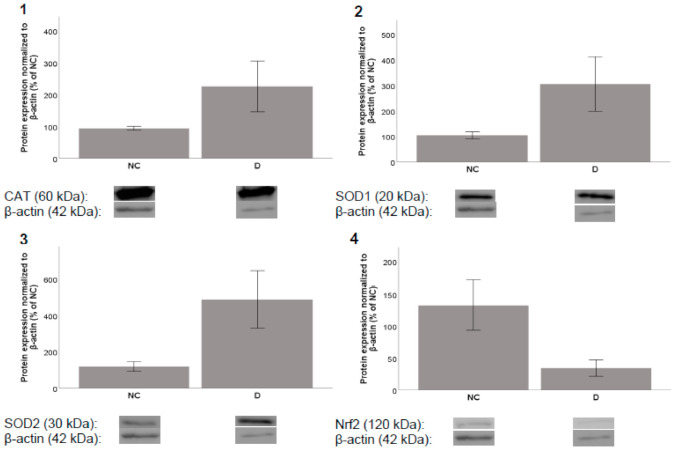
Effects of diplacone (**D**) at a concentration of 2.5 µM on the expression of (**1**) CAT, (**2**) SOD1, (**3**) SOD2, and (**4**) Nrf2 after 24 h incubation with THP-1 cells. DMSO was used as the solvent and was added as the negative control (NC). The results are expressed as the mean ± SEM and were measured in triplicate.

After incubation periods of 8 and 24 h, acteoside showed almost no influence on the expression of the selected proteins (data not shown). On the other hand, incubation with diplacone increased the level of the CAT enzyme after both 8 h and 24 h. After 8 h of incubation, diplacone treatment increased the level of SOD2 and showed a moderate effect on the expression of Nrf2. After 24 h of incubation, increases were observed in the levels of SOD1 and SOD2. Unfortunately, none of these effects were statistically significant (Figure 5 and Figure 6).

#### 2.4.2. Gene Expression

**Figure 7:**
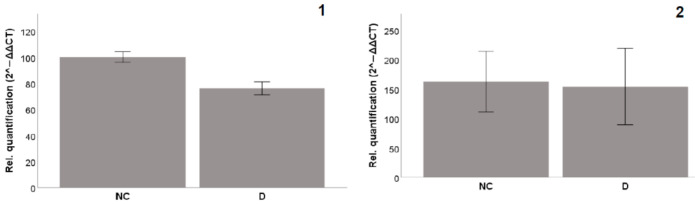
Effects of diplacone (**D**) at a concentration of 2.5 µM on the gene expression (mRNA levels) of (**1**) CAT and (**2**) SOD2 after 8 h incubation with THP-1 cells. DMSO was used as the solvent and was added as the negative control (NC). The results are expressed as the mean ± SEM for two independent experiments measured in triplicate.

To confirm the impact of diplacone, we tried to evaluate it also on the level of mRNA transcription. The results of the experiment are shown in Figure 7. For this experiment, we have chosen genes with elevated protein expression levels after 8 h of incubation. Unfortunately, we did not observe any elevation of the transcription levels of CAT or SOD2 mRNA.

#### 2.4.3. Activation of the Nrf2-ARE System

**Figure 8:**
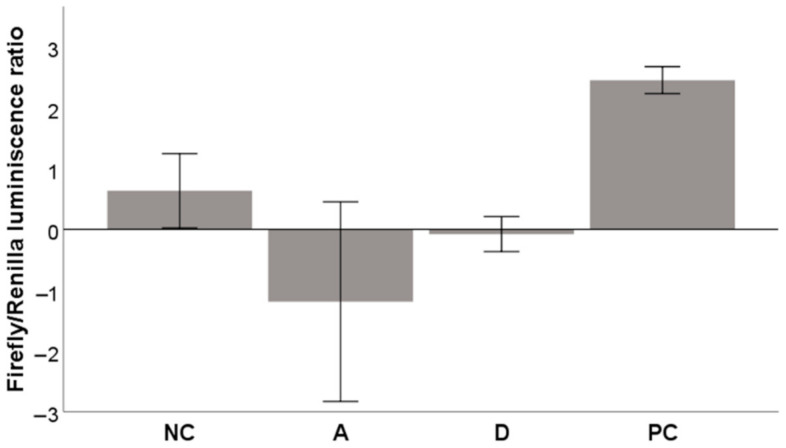
Effects of acteoside (**A**) and diplacone (**D**) at a concentration of 5 µM on the activation of the Nrf2-ARE system. The HepG2 cell model was transiently transfected with the ARE luciferase reporter vector firefly luminescence and a constitutively expressing Renilla vector. The results are expressed as the ratio of firefly to Renilla luminescence. DMSO was used as the solvent and was added as the negative control (NC). dl-sulforaphane at a concentration of 10 μM was used as a positive control (PC). The results are expressed as the mean ± SEM for two independent experiments measured in triplicate.

Next, we tried to find out if either acteoside or diplacone could support the translocation of Nrf2 to the nucleus and the activation of ARE. Figure 8 shows that the luminescence produced by the ARE luciferase reporter (normalized to Renilla luminescence) did not increase when incubated with acteoside or diplacone. This probably means that neither of these compounds activates the Nrf2-ARE system in HepG2 cells. Both compounds were tested at a nontoxic concentration of 5 µM.

#### 2.4.4. Activity of the Enzyme CAT

**Figure 9:**
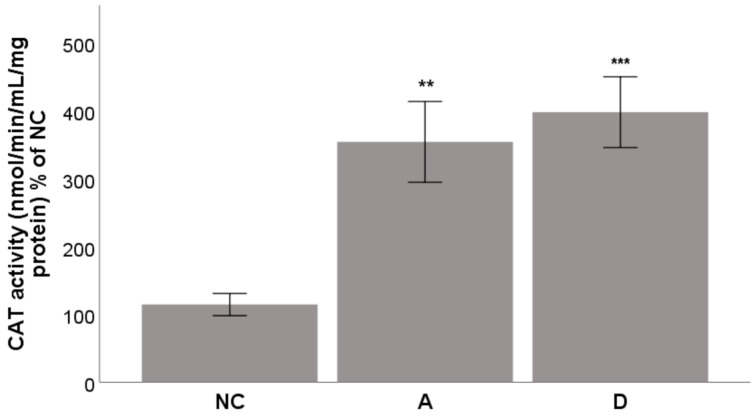
Effects of acteoside (**A**) and diplacone (**D**) at a concentration of 5 µM on the activity of the enzyme CAT. THP-1 cells were incubated with the compounds for 5 h, and then the activity of the enzyme CAT was calculated. DMSO was used as the solvent and was added as the negative control (NC). Results are expressed as the mean ± SEM for two independent experiments measured in triplicate and were statistically compared to NC (** *p* < 0.01, and *** *p* < 0.001).

Finally, one of the mechanisms contributing to the ability of acteoside and diplacone to help the THP-1 to survive under oxidative stress is an increase in the CAT activity. To discern this possibility, we carried out an experiment with an incubation time of 5 h. The results shown in Figure 9 indicate that both acteoside and diplacone increased CAT activity with statistical significance (*p* < 0.01 and *p* < 0.001, resp.). The effect of acteoside was slightly greater than that of diplacone.

Our experiments showed that the antiradical effect of acteoside was expressed more in direct scavenging of ROS in the CAA assay, but it also showed indirectly as an increase in the activity of the CAT enzyme. These two effects also appeared in the GOSP assay. Similarly, Huan et al. have found that acteoside increases the CAT activity in homogenized liver tissue [52]. Our results show that the indirect antiradical effect of acteoside is not related to increased expression of antioxidant enzymes or activation of the Nrf2-ARE system.

In contrast to the results of our experiments, Sgarbossa et al. found acteoside upregulates the expression of heme oxygenase 1 (HO-1) in both mRNA and the protein level in human keratinocyte HaCaT cells. This effect was observed after 24 h of incubation with acteoside at a concentration of 200 µM. Because the induction of the HO-1 gene is regulated primarily by Nrf2 and BACH1 transcription factors, Sgarbosa et al. also tested the influence of acteoside on the expression of these respective proteins. Whereas the Nrf2 factor activates the ARE sequence, BACH1 plays an inhibitory role. After 24 h of incubation, acteoside increased steady-state nuclear levels of Nrf2 protein and decreased the BACH1 protein levels. According to Sgarbossa et al., the antioxidant effect of acteoside is partially direct (by scavenging) and partially indirect (by activation of enzymes) [26]. We have observed only the direct scavenging effect and the indirect effect on the CAT activity, which can be due to different concentrations and cell cultures. The concentration of acteoside we used in our experiments was 40× lower than the one used by Sgarbosa et al. However. Their concentration would be obtained in vivo only with difficulty because acteoside is known for its poor bioavailability; its maximum concentration in rat plasma after peroral administration of 200 mg/kg was a mere 0.7 μM [48].

Surprisingly, diplacone showed no direct antioxidant effect in the CAA assay. On the other hand, its activity in the GOSP assay was comparable to acteoside. Diplacone also induced the expression of antioxidant enzymes, and it increased the activity of catalase in THP-1 cells. Although the results of increased expression are not statistically significant, they show the ability of diplacone to modulate the system of the antiradical defense of cells. Under specific conditions, some antioxidants may behave as pro-oxidants [53]. In the presence of heavy metals and oxygen, even some flavonoids undergo redox cycling and form ROS. A similar thing happens when flavonoids are present at high concentrations in cells [54,55]. These findings could explain why diplacone may act as a weak pro-oxidant in THP-1 cells [36] and thus activate the antioxidant defense system. In our experiment, we have observed increased Nrf2 protein levels after 8 h incubation. This accords with the overall evaluation of plant polyphenols that generate nanomolar amounts of H_2_O_2_ and thus act as activators of signaling factors [56]. However, diplacone was not shown to activate the Nrf2-ARE system, which means there may be another mechanism involved.

## 3. Materials and Methods

### 3.1. Test Compounds

The test compounds were isolated and characterized at the Faculty of Pharmacy, University of Veterinary and Pharmaceutical Sciences Brno, Brno, Czech Republic [17,18,20,21]. The purity of all compounds tested was confirmed by HPLC analysis to exceed 95% in all cases. All of the test compounds were dissolved in DMSO; the final concentration of DMSO in the cellular assays was 0.1% (*v/v*).

### 3.2. Maintenance and Cultivation of the Cell Lines

Both cell lines, THP-1 human monocytic leukemia and HepG2 human hepatoma were purchased from the European Collection of Cell Cultures (Salisbury, UK) and were cultured according to reported procedures [21,57].

### 3.3. Antiproliferative Activity

The viability of THP-1 cells was measured using the cell proliferation reagent WST-1 (Roche, Basel, Switzerland) according to the manufacturer’s manual, as reported previously [58]. The antiproliferative activity of acteoside and pomiferin in THP-1 was screened in five concentrations, ranging from 0.61 to 50 µM.

### 3.4. Cellular Antioxidant Activity (CAA) Assay

The antioxidant activity of the test compounds was measured in THP-1 cells using the method of Wolfe and Liu [59] with some modifications, as reported previously [43].

### 3.5. Glucose Oxidative Stress Protection (GOSP) Assay

The protection against glucose oxidative stress provided by acteoside and diplacone was measured in HepG2 cells using a previously reported assay with some modifications [12,13].

Briefly, the HepG2 cells were incubated in 24-well plates (100,000 cells/well) in a low glucose DMEM growth medium (Biosera, Kansas City, MO, USA; 5 mM glucose) to simulate normoglycemic conditions. High-glucose DMEM growth medium with added glucose (Sigma-Aldrich, Saint Louis, MO, USA) up to 55 mM concentration was used to create hyperglycemic conditions. The cells were incubated with acteoside, diplacone, or quercetin (used as a positive control) in a concentration of 5 µM. The solvent, DMSO, was used as a negative control (NC).

After 48 h of incubation, the cells were washed with PBS (Biosera) and further incubated for 30 min in a medium containing 10 µM 2′,7′-dichlorodihydrofluorescein-diacetate (DCFH_2_-DA; Sigma-Aldrich) dissolved in DMSO (the final concentration of DMSO in the medium was 0.1% (*v/v*)) at 37 °C. The cells were then washed again with PBS and lysed using trypsin/EDTA 1× (Biosera). The lysates were transferred into a black 96-well plate, and the fluorescence signal of the dichlorofluorescein product was measured using a FLUOstar Omega microplate reader (BMG Labtech, Ortenberg, Germany) at the wavelengths λ (ex./em.) = 485/520 nm.

The fluorescence intensity (FI) was recalculated to accord with the number of viable cells obtained from a parallel experiment with the same incubation conditions and measurement of antiproliferative activity using a WST-1 kit. The values of FI/10^6^ viable cells of the NC were assigned as 100%, and other values were referenced to these.

### 3.6. Indirect Antioxidant Activity—Modulation of Antioxidant Enzymes

#### 3.6.1. Protein Expression

The effects on protein expression of antioxidant enzymes and Nrf2 were observed in the THP-1 cell line using the method reported previously [57,60]. Briefly, the THP-1 cells were incubated in the form of floating monocytes (1 × 10^6^ cells/mL) with diplacone for 8 and 24 h. The cells were collected, and protein lysates were prepared. The lysates were then separated using SDS–PAGE, and the proteins were transferred to polyvinylidene fluoride membranes using Western blotting and visualized using antibodies and a chemiluminescent kit (Bio-Rad, Hercules, CA, USA).

Specific primary antibodies were applied: mouse anti-CAT 1:1000 (Sigma-Aldrich; product No. C0979), rabbit anti-SOD1 1:1000 (Sigma-Aldrich; product No. HPA001401), rabbit anti-SOD2 1:1000 (Sigma-Aldrich; product No. HPA001814), rabbit anti-NRF2 1:1000 (Abcam, Cambridge, UK; product No. ab137550) or mouse anti-β-actin 1:5000 (Abcam; product No. ab8226). After washing, the secondary antibodies were applied: anti-mouse IgG (Sigma-Aldrich; product no. A0168), or anti-rabbit IgG (Sigma-Aldrich; product no. A0545) at a dilution of 1:2000.

#### 3.6.2. Gene Expression

To isolate RNA and evaluate gene expression, the THP-1 cells (floating monocytes, 500,000 cells/mL) were incubated in 100 µL of a serum-free RPMI 1640 medium and seeded into 96-well plates in triplicate at 37 °C with diplacone at a concentration of 2.5 µM in DMSO.

After 8 h, the total RNA was isolated from the cells using a RealTime Ready cell lysis kit (Roche, Basel, Switzerland) according to the manufacturer’s instructions. The gene expression of CAT, SOD2, or β-actin was quantified by two-step reverse-transcription quantitative (real-time) PCR (RT–qPCR). The reverse transcription step was performed with a Transcriptor Universal cDNA Master (Roche), using cell lysate as the template. The reaction consisted of three steps: (1) primer annealing at 29 °C for 10 min, (2) reverse transcription at 55 °C for 10 min, and (3) transcriptase inactivation at 85 °C for 5 min.

A Fast Start Universal Probe Master (Roche) and gene expression assays (Applied Biosystems, Foster City, CA, USA) were used for qPCR. These assays contain specific primers and TaqMan probes that bind to an exon−exon junction to prevent DNA contamination. The parameters for the qPCR work were adjusted according to the manufacturer’s recommendations: 50 °C for 2 min, then 95 °C for 10 min, followed by 40 cycles at 95 °C for 15 s and 60 °C for 1 min. The results were normalized to the amount of ROX reference dye, and the change in gene expression was determined by the 2^−ΔΔCT^ method. Transcription of the control cells was set as 100%, and other experimental groups were multiples of this value.

#### 3.6.3. Activation of the Nrf2-ARE System

The activation of the Nrf2-ARE system in HepG2 cells was determined using an ARE reporter kit (BPS Bioscience, San Diego, CA, USA) as described previously [57]. The cells were transiently transfected for 1 h (35,000 cells/well in 96-well plates) with the ARE luciferase reporter vector (firefly luminescence) plus a constitutively expressing Renilla vector using the TransFast transfection reagent (Promega, Madison, WI, USA). After serum recovery, the cells were treated for 24 h with acteoside or diplacone at a concentration of 5 µM. As a positive control for this experiment, we used dl-sulforaphane (Sigma-Aldrich) at a concentration of 10 µM dissolved in DMSO, as recommended by ARE reporter kit. Luciferase activity from the cell lysates was detected using a dual-luciferase reporter assay system (Promega, Madison, WI, USA). Data were normalized to the Renilla luminescence.

#### 3.6.4. Activity of the Enzyme CAT

THP-1 cells (floating monocytes, 750,000 cells/mL) were incubated in 2 mL of serum-free RPMI 1640 medium and seeded into 6-well plates in triplicate at 37 °C. The cells were treated for 5 h with acteoside or diplacone at a concentration of 5 µM. The cells were then lysed, and the protein concentration was measured using the Bradford method. Then the activity of the CAT enzyme in the cell lysates was measured using a catalase assay kit (Cayman Chemical Company, Ann Arbor, MI, USA) according to the manufacturer’s instructions. The activity of CAT was expressed in nmol/min/mL/mg of proteins in the sample.

### 3.7. Statistical Analysis

Statistical analyses were carried out using IBM SPSS Statistics for Windows, software version 26.0 (Armonk, NY, USA). The data were graphed as the mean ± SEM. Comparisons between groups were made using a Mann–Whitney U test or Kruskal–Wallis test followed by pair-wise comparison with Bonferroni correction, depending on the number of experiments being compared.

## 4. Conclusions

We selected four plant phenolics previously determined to have antioxidant activity. Among these compounds, acteoside showed a direct antioxidant effect in a CAA assay. It also showed great activity in a GOSP assay. In both cases, the activity was higher than that of quercetin, the positive control. On the other hand, acteoside did show any effect on the expression of typical antioxidant enzymes or activate the Nrf2-ARE pathway. Instead, it increased only the activity of the enzyme CAT. Diplacone showed an antioxidant effect only in the GOSP assay, not in the CAA assay. This compound showed a positive effect on the expression of the enzymes CAT, SOD1, and SOD2. Again, the activity of enzyme CAT was increased.

Our results show that the antioxidant activity of compounds measured using in vitro chemical assays does not always correspond with an ability to counteract the production of ROS in cell-based systems.

## Data Availability

Not applicable.

## Sample Availability

Samples of the compounds acteoside, diplacone, morusin, and pomiferin are available from the authors.
